# Invasive phaeohyphomycosis due to Parathyridaria percutanea in a liver transplant recipient

**DOI:** 10.1186/s12879-025-12422-z

**Published:** 2025-12-22

**Authors:** Ayato Obana, Miho Akabane, Yusuke Ohashi, Neelesh Bagrodia, Austin Schenk, Sylvester Black, Kenneth Washburn, Nathan P. Wiederhold, Connie F. Cañete-Gibas, Jude Meniru, Courtney Nichols, Sajed Sarwar, Nicholas Marschalk

**Affiliations:** 1 Division of Transplantation Surgery, Department of Surgery, Comprehensive Transplant Center, The Ohio State University Wexner Medical Center, Columbus, 43210 OH USA; 2 Department of Internal Medicine, Infectious Disease, UTHealth Houston, Houston, TX USA; 3 Department of Pathology and Laboratory Medicine, Fungus Testing Laboratory, University of Texas Health Science Center, San Antonio, TX USA; 4 Division of Infectious Diseases, Department of Internal Medicine, The Ohio State University Wexner Medical Center, Columbus, OH USA; 5 Department of Hepato-Biliary-Pancreatic and Gastrointestinal Surgery, School of Medicine, International University of Health and Welfare, Chiba, Japan

**Keywords:** Parathyridaria percutanea, Liver transplantation, Opportunistic infection, Molecular diagnostics, Immunosuppression

## Abstract

**Background:**

*Parathyridaria percutanea* is a rare melanized fungus causing subcutaneous phaeohyphomycosis, previously reported only in renal transplant recipients. We report the first fatal case in a liver transplant (LT) recipient.

**Case presentation:**

A 58-year-old Somali woman developed a right foot fungating mass 10 months post-LT during intensive immunosuppression for multiple episodes of T-cell-mediated and antibody-mediated rejection (high-dose corticosteroids, antithymocyte globulin, plasmapheresis). Cultures of drainage and subsequent operative deep-tissue cultures grew *Enterococcus faecalis* and *Finegoldia* species, together with a non-sporulating filamentous mould. Despite empiric antifungals, infection progressed proximally in sporotrichoid pattern. Molecular identification using DNA sequencing of ITS and D1/D2 regions identified *P. percutanea* after three weeks. Treatment with liposomal amphotericin B/voriconazole was complicated by acute kidney injury requiring amphotericin discontinuation. Despite antifungal therapy and surgical debridement, the patient developed progressive encephalopathy and allograft dysfunction, resulting in death.

**Conclusions:**

This first fatal *P. percutanea* infection following LT highlights the importance of early molecular diagnostics for non-sporulating filamentous fungi in immunocompromised hosts and management challenges under intensive immunosuppression.

**Clinical trial number:**

Not applicable.

## Key points

First fatal liver transplant *Parathyridaria percutanea* case demonstrates diagnostic delays with conventional methods, drug interaction challenges with immunosuppression, and necessity for early molecular testing and aggressive surgical debridement.

## Introduction

Subcutaneous fungal infections typically occur following traumatic inoculation of etiologic agents into cutaneous or subcutaneous tissue, with fungi from more than 100 genera implicated as causative organisms [1]. *Parathyridaria percutanea*, first described in 2014 as *Roussoella percutanea*, is a melanized fungus belonging to the order *Pleosporales* that has emerged as a cause of subcutaneous infections [2].

Despite documented cases in renal transplant recipients [2–6], the behavior and clinical course of subcutaneous *P. percutanea* infection in other solid organ transplant populations remains unknown. Diagnosing this infection is challenging, as conventional culture methods often yield only non-specific findings of filamentous fungi [1, 7], making molecular diagnostic methods crucial for accurate identification [8]. The optimal timing and combination of antifungal therapy, particularly in the context of complex transplant immunosuppression regimens, has not been well established, as previous cases have shown variable outcomes even with appropriate antifungal therapy [2–6].

Here, we present the first case of *P. percutanea* infection in a liver transplant (LT) recipient, occurring in the context of treatment for allograft rejection with high-dose immunosuppression. This case not only expands the known host range for this pathogen beyond renal transplant recipients, but also highlights two critical aspects of management in immunocompromised hosts: the importance of early molecular diagnostic testing when conventional mycological methods yield non-specific results, and the necessity of prompt surgical debridement, which is generally considered essential for effective treatment of phaeohyphomycosis. The fatal outcome, despite appropriate antifungal therapy, underscores the need for increased awareness of this emerging pathogen in the solid organ transplant population.

## Case presentation

A 58-year-old Somali woman who had immigrated to the United States approximately 5 years earlier with a history of well-controlled type 2 diabetes mellitus and hepatitis C virus (HCV) cirrhosis underwent orthotopic LT in late 2023 at The Ohio State University Wexner Medical Center (Columbus, Ohio, USA). She had previously been treated for chronic HCV infection with the direct-acting antiviral combination sofosbuvir/velpatasvir (Epclusa) prior to LT. Her Model for End-stage Liver Disease-Sodium (MELD-Na) score was 23 at the time of transplantation from a donation after brain death donor with relevant donor/recipient [D/R] serologies: cytomegalovirus D+/R-, Epstein-Barr virus D+/R-, and donor HCV antibody and nucleic acid amplification test negative. The patient was a former smoker (0.3 pack/day for 40 years) with no history of alcohol or illicit drug use. The transplant operation was uncomplicated with an operative time of 5.4 hours, blood loss 1 liter. Her postoperative course was initially unremarkable with discharge on postoperative day 9. Post-LT immunosuppression consisted of tacrolimus (target trough 8–10 ng/mL), mycophenolate mofetil 500 mg twice daily, and a prednisone taper.

Seven months post-transplant, she developed multiple episodes of biopsy-proven T-cell mediated rejection (TCMR) and antibody-mediated rejection (AMR) requiring aggressive immunosuppression. The index liver biopsy demonstrated acute TCMR (Banff grade IIA; Rejection Activity Index 7/9) with portal microvascular inflammation and positive C4d staining, raising concern for concomitant AMR. Because liver function tests continued to worsen despite an initial course of high-dose corticosteroids, she received rescue immunosuppression with antithymocyte globulin, plasmapheresis, and intravenous immunoglobulin. Treatment included high-dose intravenous methylprednisolone (250 mg initially, followed by 500 mg daily for three doses), antithymocyte globulin (three doses of 125 mg, cumulative 4.29 mg/kg with daily CD3 monitoring), four sessions of plasmapheresis, and intravenous immunoglobulin. Her maintenance immunosuppression regimen consisted of prednisone (initially 80 mg daily with a planned taper of 20 mg every two weeks to a maintenance dose of 10 mg daily), tacrolimus 7 mg twice daily (target trough level 8–10 ng/mL), and mycophenolate sodium 360 mg twice daily.

**Fig. 1:**
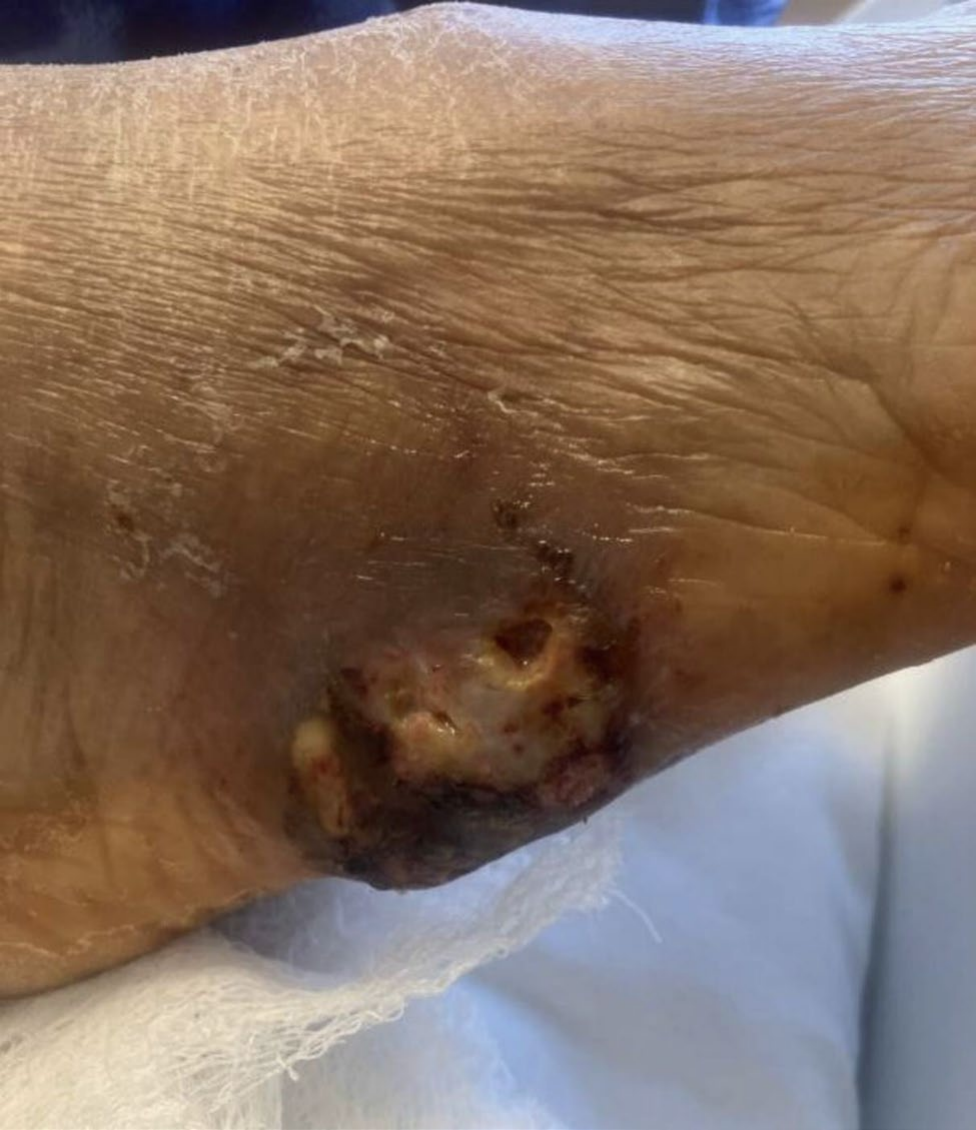
Initial presentation of the right foot wound. Clinical photograph showing a non-healing ulcerative lesion on the lateral aspect of the right foot

**Fig. 2:**
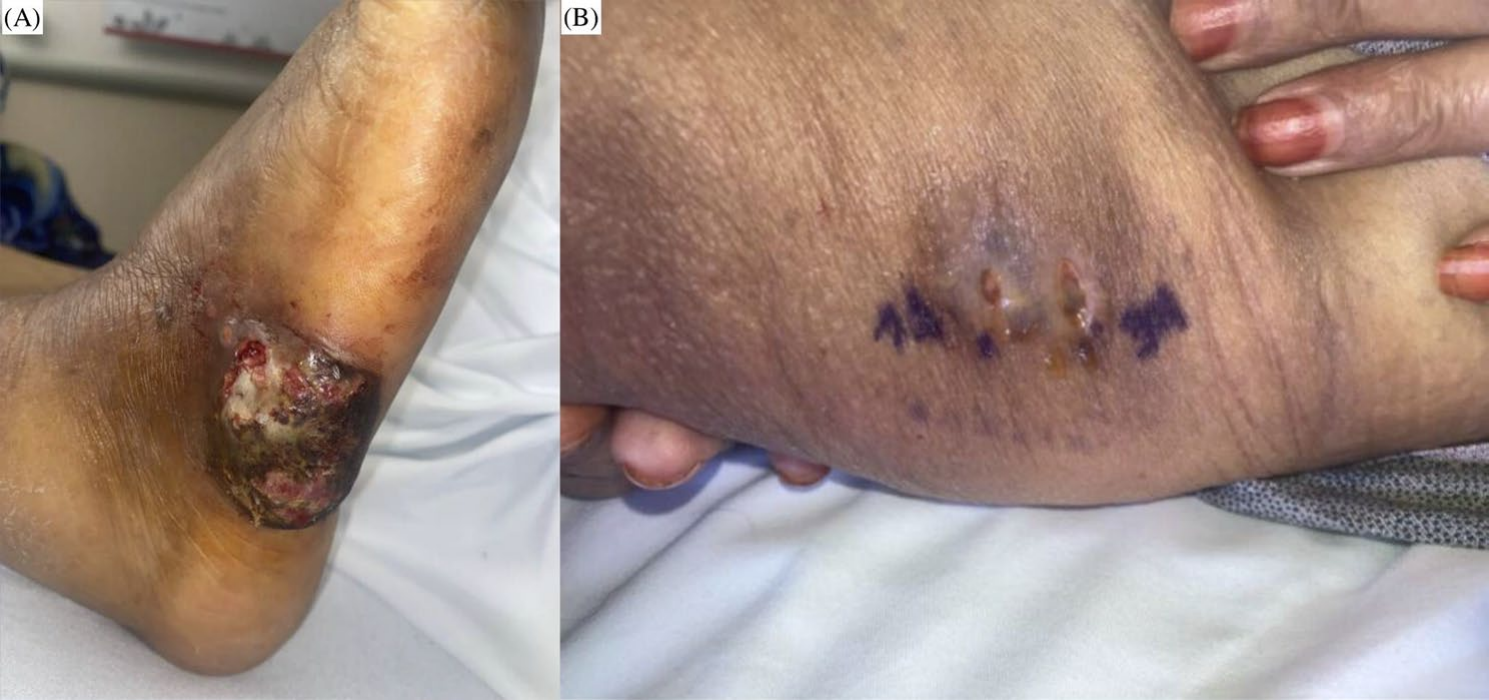
Disease progression on readmission. (**a**) progressive deterioration of the initial ulcerative lesion on the lateral right foot showing increased necrosis and purulent drainage. (**b**) sporotrichoid spread to the right posterior thigh demonstrating violaceous nodular lesions, which underwent punch biopsy of lesional tissue revealing numerous encapsulated yeasts with focal filamentous hyphal structures; culture from this biopsy again yielded a dematiaceous mould morphologically indistinguishable from the foot isolate

Around 10 months after transplant, the patient presented with an exophytic, fungating lesion involving the right foot, initially presumed to be a non-healing wound (Fig. 1) and was admitted for evaluation. She denied any history of trauma or injury to the affected area. A deep-tissue culture obtained from the ulcer base during bedside debridement initially grew *Enterococcus faecalis* and *Finegoldia species.* Although computed tomography (CT) imaging showed no definitive evidence of osteomyelitis, the presence of periostitis and chronic poor wound healing led infectious disease specialists to recommend treatment for presumptive osteomyelitis. Podiatric surgery was consulted but deferred operative incision and drainage, recommending local wound care. The patient was discharged on oral amoxicillin-clavulanate (875 mg/125 mg twice daily) with a planned 6-week course of therapy. One week after discharge, fungal culture demonstrated late growth of a non-sporulating filamentous fungus. She was readmitted approximately 2 weeks later due to this result in the context of worsening pain and wound drainage. New lesions extending proximally through her right leg and thigh in a sporotrichoid pattern were noted (Fig. 2).

**Fig. 3:**
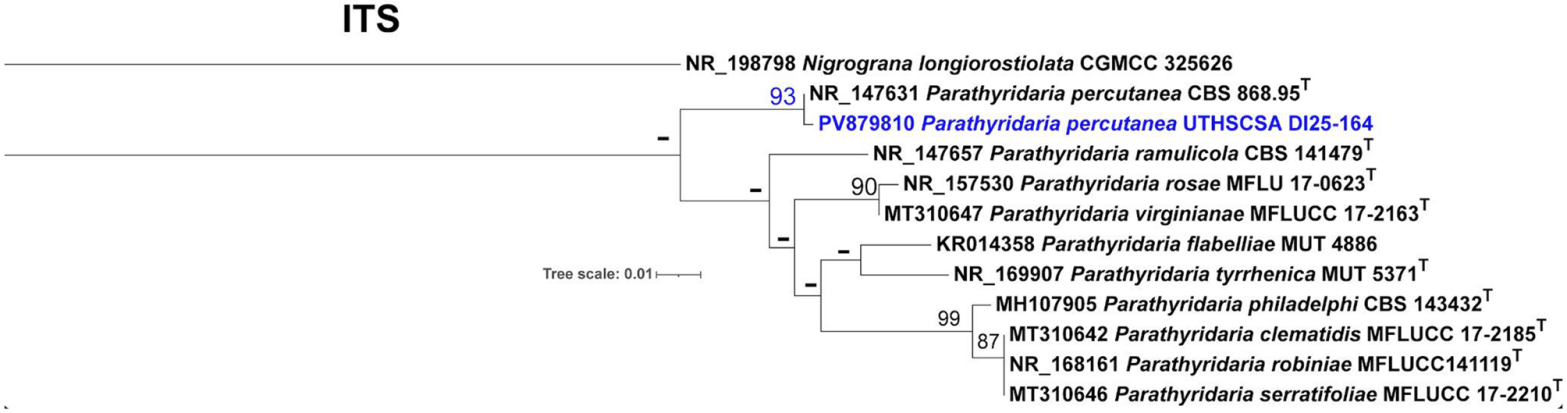
Phylogram generated from a maximum likelihood analysis of the its performed with IQ-TREE version 2. Dashes represent values lower than SH-aLRT (80%), aBayes (0.95), and BS (75%). CBS = centraalbureau voor schimmelcultures, Uppsalalaan, Utrecht, the Netherlands; CGMCC = China general microbiological culture collection Center, institute of Microbiology, Chinese Academy of sciences, Beijing, China; MFLUCC = Mae Fah Luang University culture collection, Chiang Rai, Thailand; mut = Mycotheca universitatis Tauranensis, Torino, Italy; UTHSCSA = University of Texas Health San Antonio, San Antonio, Texas USA. *T* = represent type strains. GenBank accession numbers of its and strain accession numbers are shown before and after the name

Upon readmission, punch biopsies of the right thigh lesions were obtained, and fungal culture from biopsy yielded a mould that, on review by our microbiology laboratory, was morphologically indistinguishable from the original foot isolate. Magnetic Resonance Imaging of the right foot suggested possible osteomyelitis of the fifth metatarsal, and right lower extremity duplex was negative for acute deep vein thrombosis. The patient subsequently underwent surgical debridement, with removal of necrotic skin and subcutaneous tissue down to viable bleeding tissue; no gross bone resection was performed, and source control was considered macroscopically adequate at the time of surgery, with intraoperative deep-tissue cultures growing filamentous fungi. Notably, the initial fungal culture from the index admission could not be identified by our laboratory’s routine methods and was sent to the Fungus Testing laboratory at the University of Texas Health San Antonio (UTHSCSA) for identification. The isolate, UTHSCSA DI25-164, was identified by combined phenotypic characterization and DNA sequencing of the ITS region (GenBank accession number PV879810) and the D1/D2 domains of the large subunit gene (*LSU*) of the rDNA (GenBank accession number PV879912). Based on BLASTn searches in NCBI’s GenBank nucleotide database [9], using ITS, the closest hits showed the highest similarity to *Parathyridaria percutanea* CBS 868.95 GenBank NR_147631 at 99%; *Parathyridaria ramulicola* CBS 141479 at 93%. Closest hits using *LSU* were *Parathyridaria percutanea* CBS 868.95 GenBank NR_147631 at 100% and *Parathyridaria ramulicola* CBS 141479 at 96%. The definitive identity of the organism as *Parathyridaria percutanea* was confirmed by phylogenetic analysis using ITS, wherein, the isolate clustered with *Parathyridaria percutanea* CBS 868.95 with high branch support (SH-aLRT = 86.5%; Bootstrap value = 93%) and phylogenetically distant from the other species in the genus (Fig. 3) [2].

Based on the initial fungal findings with identification pending, the patient was started on liposomal amphotericin B (5 mg/kg/day). Approximately one week later, following species identification, voriconazole (6 mg/kg every 12 hours for two doses, followed by 4 mg/kg every 12 hours) was added to the regimen. Voriconazole trough concentrations were monitored and remained within the therapeutic range (2–5 µg/mL). However, the patient developed worsening acute kidney injury, necessitating discontinuation of liposomal amphotericin B. Despite these treatment modifications, her condition deteriorated with progressive encephalopathy and persistent liver allograft dysfunction. The sporotrichoid nodular lesions along the right lower extremity and thigh persisted, with marked edema of the right leg, raising concern for ongoing lymphatic spread of cutaneous phaeohyphomycosis. CT imaging of the chest and sinuses showed no evidence of invasive fungal disease. Blood cultures remained negative throughout the admission. The patient was ultimately found unresponsive and expired despite resuscitative efforts. While disseminated fungal infection remained a clinical concern, particularly in the setting of worsening multiorgan dysfunction, it could not be definitively confirmed as cause of death in the absence of autopsy.

## Discussion

This case highlights two significant findings regarding *P. percutanea* infection in solid organ transplant recipients. First, this case adds to the limited literature on *P. percutanea*, which has previously been reported only in renal transplant recipients [2–6], and suggests that liver transplant recipients may also be at risk. Second, this case illustrates the importance of timely molecular diagnostic methods and prompt surgical management in immunocompromised hosts. Although initial wound and deep-tissue cultures yielded a dematiaceous mould, species-level identification required nearly three weeks, during which time the infection spread from a localized foot wound to sporotrichoid nodular lesions along the right lower extremity despite systemic antifungal therapy. Surgical debridement achieved macroscopic removal of necrotic soft tissue but did not include bone resection, and persistent edema and nodularity of the limb raised concern for residual infection and incomplete source control. In the setting of ongoing rejection, intensive immunosuppression, and evolving multiorgan dysfunction, we cannot quantify the exact contribution of phaeohyphomycosis to the fatal outcome; however, the clinical course suggests that earlier diagnosis and more aggressive local control may be important considerations in similar cases.

**Fig. 4:**
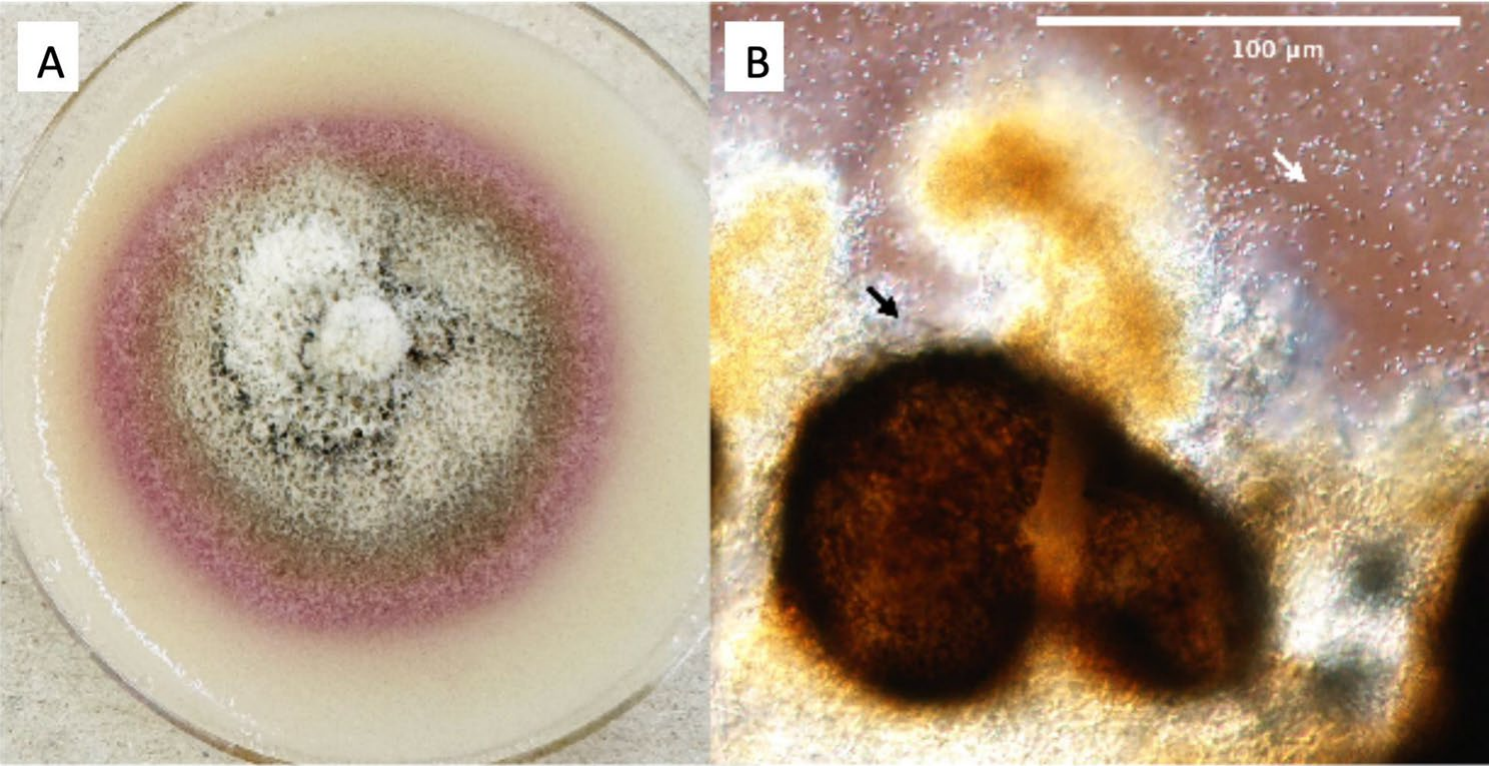
*Parathyridaria percutanea* (UTHSCSA DI25-164). (**A**) colony on potato flakes agar, 21 days at 25 °c; (**B**) pycnidium (black arrow), conidia (white arrow)

*P. percutanea* formerly known as *Roussoella percutanea*, is a coelomycetous fungus in the family Thyridariaceae in the order Pleosporales, characterized by conidia or asexual propagules enclosed within a cavity called pycnidium (pl pycnidia) and includes poorly sporulating or nonsporulating dematiaceous fungi (Fig. 4). *Parathyridaria* spp. are generally plant saprobes, with *P. percutanea* being the only species reported as an opportunistic human pathogen [10]. The joint guidelines of the European Society of Clinical Microbiology and Infectious Diseases Fungal Infection Study Group and the European Confederation of Medical Mycology on the management of phaeohyphomycosis in subcutaneous, bone and joint infections recommends surgical resection as well as azoles (itraconazole, voriconazole, or posaconazole) or amphotericin B [11].

The occurrence of *P. percutanea* infection in a LT recipient represents a significant expansion of its known host range. Previous cases have been exclusively documented in renal transplant recipients with maintenance dose immunosuppression [2–6]. In contrast, our patient’s infection developed in the context of intensified immunosuppression for allograft rejection, including high-dose corticosteroids, antithymocyte globulin, and plasmapheresis, which likely contributed to both acquisition and rapid progression of the infection. Previous reports have suggested that a combination of effective antifungal therapy and surgical intervention provides optimal treatment for *P. percutanea* infections [3], with triazole antifungals demonstrating the highest in vitro activity [2, 3]. However, several factors likely contributed to the fatal outcome in our case. First, delayed surgical intervention allowed the infection to progress from a localized lesion to extensive sporotrichoid spread before definitive treatment was initiated. Second, while triazole antifungals such as voriconazole are frequently co-administered with tacrolimus, their interaction requires careful monitoring [12]. In this patient, tacrolimus trough levels were monitored and adjusted according to our institutional protocol, and voriconazole trough concentrations were maintained within the therapeutic range, as described above. Third, the need for molecular identification at a reference laboratory resulted in a significant delay between initial culture and definitive diagnosis. Additionally, our patient, a US immigrant from Somalia, adds to prior human cases reported from India, Curaçao, and Somalia [3, 8], suggesting that P. percutanea has a broad geographic distribution. Although the exact timing of her last travel outside the United States was not documented, her status as a recent immigrant raises the possibility of remote environmental exposure to the organism prior to transplantation, consistent with the hypothesis of latent subcutaneous infection later reactivated under immunosuppression. This case, like previous reports, suggests that relatively dormant fungi may remain subcutaneously latent for years or decades before becoming clinically active following immunosuppression [1]. While subcutaneous mycoses typically result from traumatic inoculation [1], the extended and variable latency period explains why most patients, including our case and previously reported cases of *P. percutanea* infection, cannot recall specific trauma to the affected sites [2–6].

The diagnostic challenges encountered in this case underscore the critical importance of molecular methods in identifying rare fungal pathogens. The initial fungal culture yielded only non-specific findings of a non-sporulating filamentous fungus, a common limitation in conventional mycological diagnostics [1, 2, 7]. Despite the presence of clear clinical progression, definitive identification required nearly three weeks of processing at a reference laboratory using combined phenotypic characterization and DNA sequencing of the ITS and *LSU*. Given that conventional mycological methods can identify only a small fraction of existing fungal species [10, 13], early referral for molecular diagnosis becomes crucial, particularly in immunocompromised hosts where delayed targeted therapy may result in disease progression. The fatal outcome in our case, where the infection progressed from a localized foot wound to extensive involvement of the lower extremity during the period of diagnostic uncertainty, emphasizes the need for expedited molecular testing in similar clinical scenarios.

Several clinical and microbiologic features support a pathogenic role for *P. percutanea* in this case, including its repeated isolation from deep wound tissue and the development of progressive sporotrichoid nodules with histopathologic evidence of fungal infection in the thigh biopsy. However, the index wound cultures were polymicrobial, and we did not perform semi-quantitative histology or qPCR-based pathogen load quantification. Thus, while these data strongly implicate *P. percutanea* as a key driver of the sporotrichoid dissemination, we cannot exclude a contributory role of co-isolated bacterial pathogens such as *Enterococcus faecalis* and *Finegoldia* species or definitively establish *P. percutanea* as the sole or dominant pathogen.

Based on our experience with this case, several key recommendations can be made for managing suspected *P. percutanea* infections in solid organ transplant recipients. First, early initiation of molecular diagnostic testing is crucial when conventional cultures yield non-specific filamentous fungi, particularly in immunocompromised patients. While awaiting identification, empiric antifungal therapy should be considered, but careful attention must be paid to drug-drug interactions, especially in LT recipients where azole antifungals may significantly impact tacrolimus levels as well as contribute to potential hepatotoxicity. Second, a high index of suspicion for similar dematiaceous fungal infections, including subcutaneous phaeohyphomycosis due to melanized moulds such as *P. percutanea*, should be maintained in transplant recipients with progressive subcutaneous infections, particularly those with a history of international travel or immigration from regions where previous cases have been reported [3, 8]. Finally, given the potential for rapid progression in highly immunosuppressed patients, early surgical debridement should be considered as part of the initial management strategy, even before definitive identification is available [3].

In conclusion, this case represents the first documented fatal *P. percutanea* infection in a LT recipient and highlights several important clinical lessons. The convergence of multiple risk factors - intensified immunosuppression for rejection, delayed molecular identification, and complex drug interactions limiting antifungal options - likely contributed to the adverse outcome. For clinicians managing immunocompromised transplant recipients with suspicious fungal infections, this case emphasizes three critical points: the importance of early molecular diagnostic testing when conventional cultures are non-diagnostic, the need to consider drug-drug interactions when selecting antifungal therapy, and the potential value of early surgical intervention. As the spectrum of opportunistic fungal infections continues to expand in the transplant population, maintaining awareness of emerging pathogens like *P. percutanea* and establishing protocols for expedited molecular testing may help improve outcomes in similar cases.

## Data Availability

No datasets were generated or analysed during the current study. All data supporting the findings of this case report are contained within the article and its supplementary material.

## Abbreviations

AMR: Antibody-mediated rejection
CT: Computed tomography
HCV: Hepatitis C virus
ITS: Internal transcribed spacer
LT: Liver transplant
LSU: Large subunit gene
MELD-Na: Model for End-stage Liver Disease-Sodium
TCMR: T-cell mediated rejection
UTHSCSA: University of Texas Health Science Center San Antonio
